# The Sensitivity Improvement Characterization of Distributed Strain Sensors Due to Weak Fiber Bragg Gratings

**DOI:** 10.3390/s20226431

**Published:** 2020-11-11

**Authors:** Konstantin V. Stepanov, Andrey A. Zhirnov, Anton O. Chernutsky, Kirill I. Koshelev, Alexey B. Pnev, Alexey I. Lopunov, Oleg V. Butov

**Affiliations:** 1 Bauman Moscow State Technical University, 2-nd Baumanskaya 5-1, 105005 Moscow, Russia; a.zh@bmstu.ru (A.A.Z.); chernutsky.a@bmstu.ru (A.O.C.); koshelev@bmstu.ru (K.I.K.); pniov@bmstu.ru (A.B.P.); 2 Kotelnikov Institute of Radioengineering and Electronics of RAS, Mokhovaya 11-7, 125009 Moscow, Russia; aley@mail.ru (A.I.L.); obutov@mail.ru (O.V.B.)

**Keywords:** weak fiber Bragg gratings, distributed fiber optic sensor, phi-OTDR

## Abstract

Weak fiber Bragg gratings (WFBGs) in a phase-sensitive optical time-domain reflectometer (phi-OTDR) sensor offer opportunities to significantly improve the signal-to-noise ratio (SNR) and sensitivity of the device. Here, we demonstrate the process of the signal and noise components’ formation in the device reflectograms for a Rayleigh scattering phi-OTDR and a WFBG-based OTDR. We theoretically calculated the increase in SNR when using the same optical and electrical components under the same external impacts for both setups. The obtained values are confirmed on experimental installations, demonstrating an improvement in the SNR by about 19 dB at frequencies of 20, 100, and 400 Hz. In this way, the minimum recorded impact (at the threshold SNR = 10) can be reduced from 100 nm per 20 m of fiber to less than 5 nm per 20 m of fiber sensor.

## 1. Introduction

Distributed fiber-optic monitoring systems based on phase-sensitive optical time-domain reflectometers (phi-OTDR) are gaining popularity. Their indisputable advantages include the ability to control objects of considerable length (pipelines, perimeters) automatically, allowing real-time detection of the magnitude and location of impacts on the sensor. They operate by analyzing backscattered radiation from a pulse of a highly coherent radiation source. In this case, the sensor has a high sensitivity in analyzing the phase deviations of the received signal. The received signal—the reflectogram—remains unchanged until the moment of any impact on the sensor. For example, the system can receive information about vibration effects, the analysis of which can be used to register a person’s passages, excavations, vehicle movements, and other activities. The history of such systems began in the 1990s with the works of the Taylor group [1,2,3,4,5]. Gradually, such systems have become more common given the development of fiber optics. Canadian scientists have paid much attention to polarization effects and signal processing using wavelets [6,7,8]. Scientists from Spain, led by H.F. Martins, worked on various aspects of the system’s operation, including implementing them, increasing the sensor length using Raman amplifiers, and measuring temperature using chirped pulses [9,10,11,12]. In Russia, several scientific groups are also engaged in this issue, with particular attention to the device’s principles and limitations, the possibilities of using it as a temperature sensor, and the development of principles for restoring the phase of the recorded signal [13,14,15,16,17,18,19,20]. Many results have found practical applications in commercial systems, including in the oil and gas industry [21,22]. In the last 10 years, many scientific papers have been published that comprehensively considered methods of increasing the range of systems operation, signal processing, various applications, and tracking the frequency offset of a laser source [23,24,25,26]. Some of these works were devoted to numerically measuring the strain exerted on the fiber. However, the physical nature of the signal formation by a phase-sensitive reflectometer based on Rayleigh scattering makes signal changes nonlinear and nonsinusoidal, including with monotonic external influences. A significant step toward explicitly representing the relationship between external influences and signal changes would be to modify sensors with WFBGs. Patents for such a sensor device first appeared a long time ago [27], but they have only received practical implementation relatively recently [28,29,30]. Before this point, gratings at different wavelengths were mainly surveyed, or wavelength shifts were monitored [31,32]. In such a case, only characteristics (temperature or strain) of the WFBG contact area can be measured. A system with 843 FBGs in one fiber was experimentally demonstrated [32]. Since 2015, articles about WFBGs with phi-OTDR interrogation have become regular. Scientific groups in China carried out the first works surveying WFBGs, similarly to phase-sensitive reflectometry. A group from Shandong and Wuhan Universities experimentally showed a work of setup with 500 WFBGs. They achieved the pressure detection limit of 0.122 Pa in a frequency range from 450 to 600 Hz [33]. Scientists from Nanjing University in a few publications showed 11 dB enhancement in SNR with the help of WFBGs and described a quantitative strain measurement with 6.2 nε maximum error [33,34,35,36,37]. They also theoretically showed thelimitations of WFBG’s quantity in one sensor due to attenuation on an array of them [35,36]. Since then, many studies have been carried out. Works have been presented on the possibility of restoring the impact phase by using Mach–Zehnder [38] and Michelson [39] interferometers in the recording branch. It can achieve a fiber length variation sensitivity of 117 pm/√Hz with a distance between WFBGs equal to 99 m [38]. Investigations of vast arrays of WFBG were carried out in a laboratory: setup with 964 WFBGs for nε-scale deformation measurements [40], 90 WFBGs helped to detect 14.63 nm deformations of 50 m fiber between neighboring reflectors [41]. Parallel measurements using traditional sensors were carried out: phi-OTDR system with 660 WFBGs was compared to geophones and showed good agreement in signal shape [42]. Simultaneously, the measurement results in such systems have made it possible to obtain deformation values, including in microstrains—that is, showing the elongation of the fiber [40]. RMS measurement error of fewer than 1.32 n*ε* was demonstrated in a frequency range from 8 to 1 kHz [43]. This is in agreement with the results of the other group: static strain resolution of 1.89 nε and dynamic resolution of 97.5 pε/√Hz over 1 Hz [44]. Possibilities have been investigated for increasing the sensor interrogation rate three times using measurements at several wavelengths generated by AOMs with different modulation frequencies [45]. Additionally, the first studies were carried out to compare the recorded signals of a fixed frequency and the amplitude on systems with WFBGs and with a conventional phase-sensitive reflectometer in the spectral region. Exceeding of the peak level over the noise level was improved by 27 dB in an experiment with a 1 kHz impact produced by PZT [46]. In 2019, the practical application of such a system was shown for the first time. Phi-OTDR with WFBGs was used for subway monitoring. This can detect intrusion events, e.g., illegal excavator work [47]. The first steps were done to the next generation sensors, based on scattering dots. These can be fs-inscribed into fiber, which is technological in comparison to WFBG producing technology. The first experiments were conducted to show the possibility of signal improvement, similar to WFBG [48]. It was also demonstrated that a combination of frequency and phase domain time-gated coherent reflectometry methods based on a sensor with artificial reflectors can measure the distance between them [49]. For spacing between dots in fiber 9.84 ± 0.04 m, the measurement error was 0.16 μm. But this technology still needs improvement due to the instability of dot’s reflection coefficients produced in one fiber.

This study was a theoretical analysis and experimental comparison of the growth in signal-to-noise ratio at the same fiber deformations in systems based on a classical phase-sensitive reflectometer scheme versus a scheme using WFBG. For this purpose, it is necessary to disassemble the signal-formation process and determine the primary sources of noise. The relevance of this problem is due to the peculiarity of signal registration by a real system in the field, which depends on a large number of parameters, including the type of fiber, the cable design, and the characteristics of the soil in which the cable is laid. On average, for a classical scheme with a phase-sensitive reflectometer, the passage and the performance of any manual work (such excavation) is believed to be detectable at a distance of no more than 5 m from the sensor cable [50,51,52]. At the same time, due to technological features, laying the sensor cable within just such a distance from the monitoring object is often difficult or impossible, which results in the distance from the sensor to the potential impact site exceeding the indicated 5 m, so that the event is not registered. To solve this problem, the minimum detectable level of cable deformation must be lowered. This can be done, for example, by adding hydrogel to the cable for better transmission of vibration effects directly to the fiber, thus increasing the sensor’s sensitivity. Nevertheless, even if the sensitivity of the cable itself is improved, some sources of system noise cannot be eliminated. In a phase-sensitive reflectometer, the primary sources of noise are:

1. Frequency instability of the laser source, which affects the stability of the interference backscattered signal’s formation;
2. Signal-spontaneous noise from the preamplifier (beats of the signal with spontaneous emission of the preamplifier within the optical filter’s transmission), which is several orders of magnitude higher than the preamplifier’s spontaneous-spontaneous noise (the beats of the spectral components of the preamplifier’s spontaneous emission within the optical filter’s transmission) due to the use of narrow-band filters in the receiving line [53];
3. The noise of the photodetector module.

In this paper, we consider the influence of low-reflective fiber Bragg gratings on these components of the parasitic noise. Thus, for example, the presence of a WFBG can remove one main source of noise: the erbium preamplifier.

## 2. Theory

### 2.1. Signal Formation in a Phase-Sensitive Reflectometer

**Figure 1:**
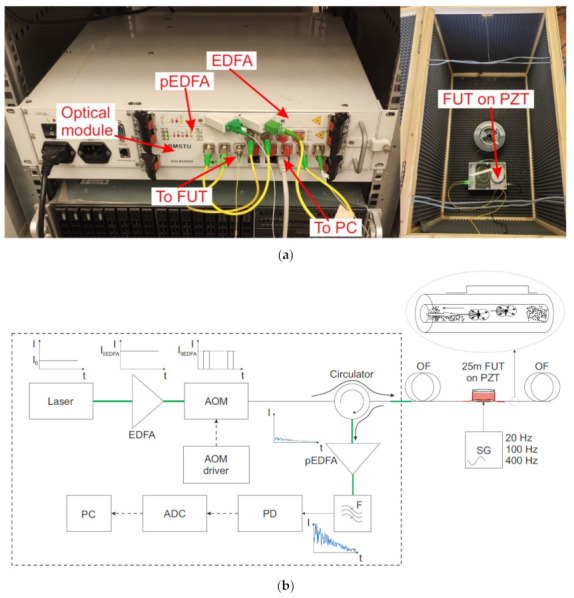
Photo and scheme of an experimental phase-sensitive reflectometer for determining a sensor system’s sensitivity based on Rayleigh backscattering. Laser: radiation source, EDFA: optical erbium amplifier, OM: optical modulator, OF: optical fiber, FUT: fiber section for testing impacts, pEDFA: optical erbium preamplifier, F: optical filter, PD: photodiode, ADC: analog–digital converter, PC: personal computer. Thin solid lines—fiber optic connections inside the optical module. Bold solid lines—fiber connections outside the case. Dashed lines—electrical connections. (**a**): photo of setup; (**b**): scheme of setup.

A phase-sensitive reflectometer is a device operating based on the effects of Rayleigh scattering. The scheme is shown in Figure 1. The radiation from a highly coherent source (a laser) is brought to the required power in an amplifier (EDFA). Next, probing pulses are formed in the optical modulator (OM), which are directed through the circulator in the forward path to the sensor fiber, with the scattered radiation from the reverse path is directed to the preamplifier (pEDFA) to increase the power. The preamplifier’s spontaneous emission is suppressed with an optical filter (F). Next, the radiation is registered by a photodetector (PD), digitized on an analog–digital converter (ADC), and sent for processing on a computer (PC). Information about an impact on the fiber is extracted from the instability of the reflectograms, specifically the intensity distribution of the backscattered radiation along the sensor cable.

**Figure 2:**
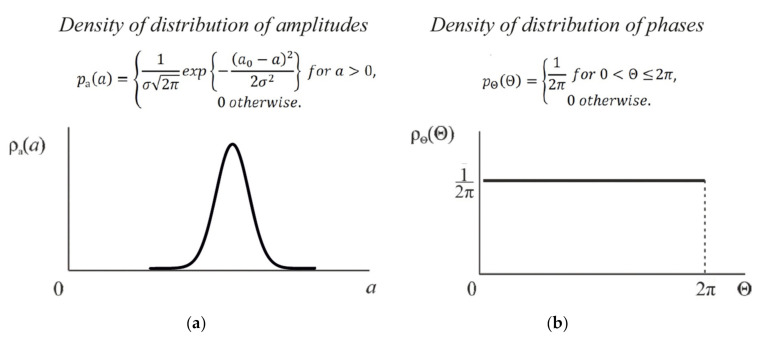
The density of the amplitude (**a**) and phase (**b**) distributions of backscattered radiation.

If the radiation from the source of the probing signals has a coherence length much longer than the pulse duration, then the backscattered waves will not be added as the integrated powers, but as the amplitudes which take the phases into account. In this case, the scattering centers are any inhomogeneities in the fiber core, which are located in the glass structure chaotically and with high density. As a result, the distribution of the backscattered waves’ amplitudes obeys Gauss’s law, and the distribution of phases is uniform over the interval from 0 to 2π, as shown in Figure 2.

The signal level in this device is determined by the parameters of the probing pulse: the peak power and duration. The power from above is limited by the threshold for the appearance of nonlinear effects, primarily modulation instability [17,54], and the duration is limited by the spatial resolution necessary for the device, which is the half-width of the pulse in the fiber.

The reflectogram obtained from the device has a jagged appearance, varying from maximum to minimum, depending on how the interference of backscattered waves occurs in each area of the sensor. The resulting form of the reflectogram remains stable until the scattering centers’ positions change. When they are displaced under external influence, the form of the reflectogram changes. On this basis, one can discuss the activity in this area of the sensor.

**Figure 3:**
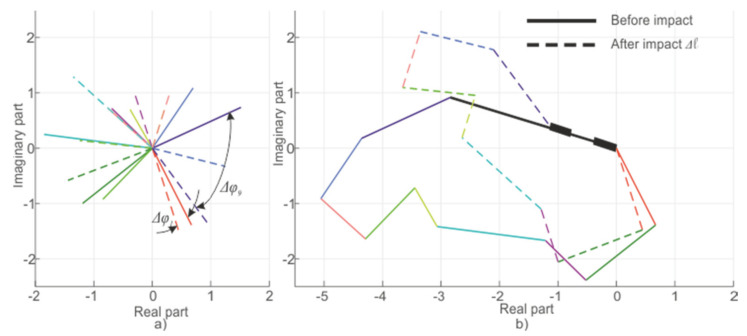
Scheme of signal generation in a phi-OTDR: components (**a**) and summation (**b**). A line of each color represents one scattered wave: the length is equal to the amplitude and the angle is equal to the phase.

In the case of a system based on Rayleigh scattering, the signal formation can be represented as a result of the backscattered waves being added from all of the inhomogeneities within the half-width of the pulse duration. Since each wave has a random amplitude with a Gaussian probability density distribution, the change in the phase of each wave due to the scattering center’s displacement makes a nonlinear contribution to the change in the final signal recorded by the system. This process is shown in a simplified form in Figure 3, which, for clarity, shows the result of adding 10 waves before (solid line) and during impact Δ*l* on the fiber (dotted line). The colors show the waves from each center of scattering.

Mathematically, this can be expressed as the sum of *n* backscattered waves from inhomogeneities within the pulse half-width that have a Gaussian distribution of amplitudes

(1) $$p_{E}(a)=\left\{\begin{array}{c} \frac{1}{\sigma \sqrt{2\pi }}\exp\left(-\frac{{\left(a-a_{0}\right)}^{2}}{2\sigma^{2}}\right)\;for\;a>0,\\ 0\;otherwise, \end{array}\right.$$

(2) $$E^{2}={\left|\sum_{m=1}^{n}E_{m}\exp(-kr_{m})\right|}^{2},$$

(3) $$\Delta \varphi =arg\left(\sum_{m=1}^{n}E_{m}exp\left(-\frac{2\pi l_{m}}{\lambda }\right)\right)-arg\left(\sum_{m=1}^{n}E_{m}exp\left(-\frac{2\pi l\prime_{m}}{\lambda }\right)\right),$$

where *a* is the amplitude of the scattering center, *a*_0_ is the expected value of the amplitude, *k* is the wavenumber of the backscattered wave, *p_E_* is the function describing the probability density of the wave amplitude, and *σ* is the Gauss distribution parameter. Additionally, *E_m_* is the amplitude of the *m*-th scattering center out of *n* included in the pulse half-width, *l_m_* is the distance from the radiation source to the *m*-th scattering center, *λ* is the current wavelength of the system’s laser source, and Δ*φ* is the signal phase change caused by fiber deformation.

This shows that the phase changes of each backscattered wave will differ when the fiber section is linearly stretched: all phase changes from the nearer sections will be added to the phase change in the distant sections. As a result, the increase in the phase change will increase for different sections in different ways, and the signal will not change according to a simple sinusoidal law but a complex harmonic law.

**Figure 4:**
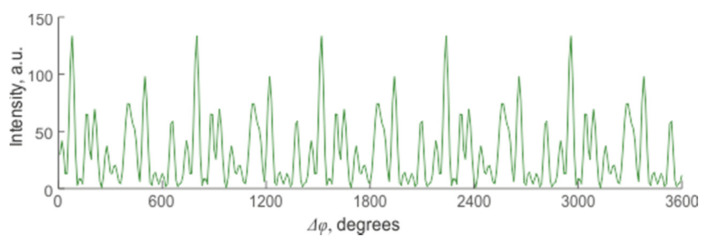
Change in the resulting intensity of the backscattered signal depending on the amount of deformation in a system with many randomly located reflectors.

This expression shows that the displacement of each center of scattering contributes to the change in the interference result and the signal’s magnitude from the sensor area. Yet, it is impossible to predict whether this change will lead to an increase or decrease in the signal or to predict its monotony and periodicity. One of the options for changing the resulting intensity is shown in Figure 4.

### 2.2. Signal Conditioning in a WFBG-Based System

**Figure 5:**
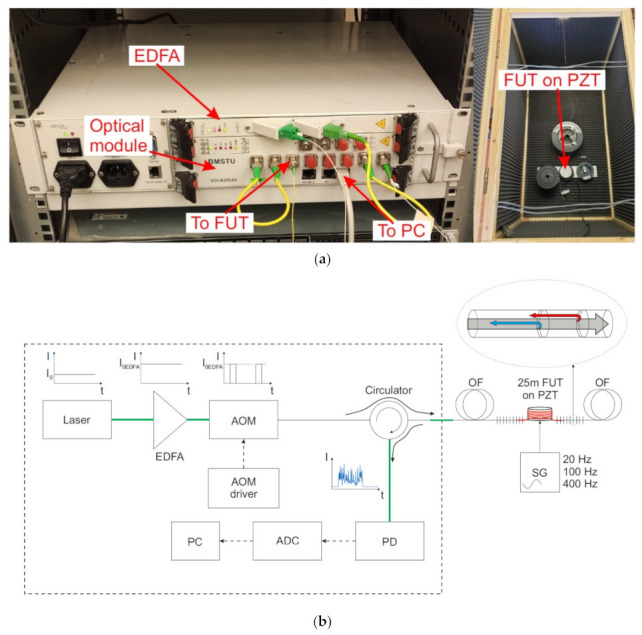
Photo and scheme of a WFBG-based system for experimenting to define sensitivity. Laser: radiation source, EDFA: optical erbium amplifier, OM: optical modulator, OF: optical fiber, FUT: fiber section for testing, PD: photodiode, ADC: analog-to-digital converter, PC: personal computer. Thin solid lines—fiber optic connections inside the optical module. Bold solid lines—fiber connections outside the case. Dashed lines—electrical connections. (**a**): photo of setup; (**b**): scheme of setup.

A sensor based on a fiber with periodically applied WFBG can be used to obtain a higher-intensity signal from the sensor. The technology allows creating such structures directly during the extraction of fiber, which makes it convenient and inexpensive [28,30]. The reflection coefficient of such WFBGs can be fractions of a percent or less, while their relative amplitude spread in the array will be no more than 15% [55]. A scheme of the system based on WFBGs is shown in Figure 5. The operating principles are similar to those of a phase-sensitive reflectometer, but the signal back-reflected from the sensor fiber has significantly higher power, and a preamplifier and a filter are not needed to suppress spontaneous emission.

To interpret the received data, it can be assumed that each section between adjacent WFBGs generated a separate signal. Notably, signal interference will be observed only in the case when the pulse length *L_pulse_* is twice the distance of that between WFBG1 and WFBG2 *L* because to observe the interference of reflected signals, the pulse reflected from WFBG2 must have time to return and interact with the signal reflected from WFBG1. Provided that all WFBGs are located at the same distance, then the length of the scanning pulse must meet the following condition to obtain a clear interference pattern from two adjacent WFBGs:

(4) $$2L<L_{pulse}<4L,$$

**Figure 6:**
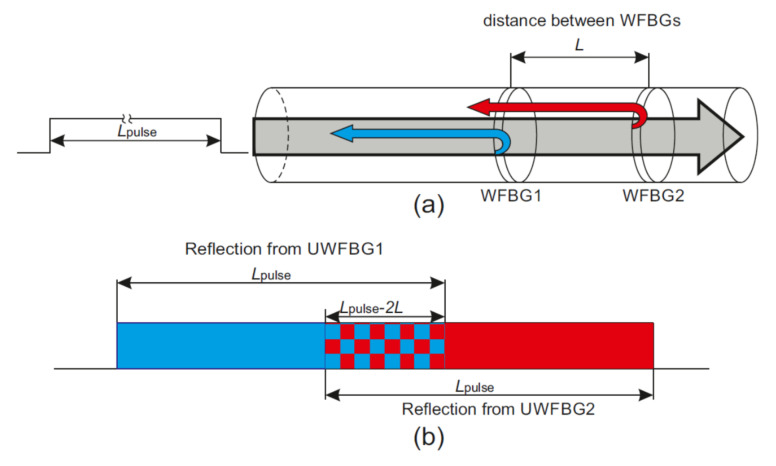
Backscattered signal formation from the WFBG pair. (**a**): Pulse reflection from neighboring WFBGs; (**b**): Scheme of reflected light interference.

In this case, the pulse duration should be less than 4*L*, so that reflection from more distant gratings will not affect the interference pattern. The interference of reflected signals from two neighboring WFBGs is demonstrated in Figure 6.

Thus, a sensor with periodically applied WFBGs can be compared to a sensor in a conventional reflectometer with scattering centers located with a large and strict periodicity and the same reflection coefficients. In comparing the back-reflected power, a conventional reflectometer, as mentioned earlier, will have a value of 10^−4^%, and a modified one will have a value of *R*—that is the WFBG reflection coefficient. In other words, the value of the useful signal can be several orders of magnitude higher, which allows it to be registered without an erbium preamplifier. The only limitation on the WFBG reflection level is the number of sections on the scanning line [35,36]. Importantly, when scanning a sensor line, it must be possible to detect signals from distant sections.

Thus, the use of sensors with WFBGs should theoretically reduce the noise level and allow impacts on a sensor with a much lower intensity to be recorded than is possible with conventional fiber. The experimental determination of this quantity using laboratory facilities is described in the next section.

**Figure 7:**
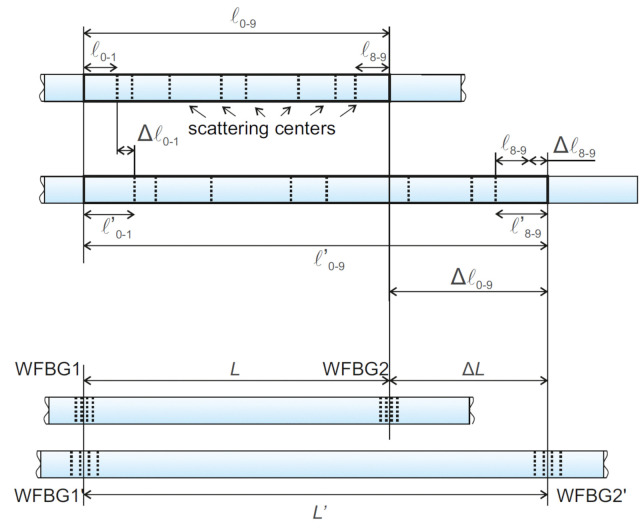
Scheme for changing the distance to the reflector in a phase-sensitive reflectometer based on backward Rayleigh scattering (top) and using WFBGs (bottom).

It is also necessary to note the peculiarity of the signal formation from lines with WFBGs in the case of classical phi-OTDR. The difference in deformations is shown in Figure 7. With a uniform stretching of the section for a phi-OTDR, the distance between each pair of adjacent scattering centers increases by concrete value for this pair. For a sensor with WFBGs, the equivalent stretch of the fiber section will affect the phase difference only between the backscattered waves from WFBG*_i_* and WFBG*_i+_*_1_, which makes the dynamics of the fiber-deformation process easy to understand.

**Figure 8:**
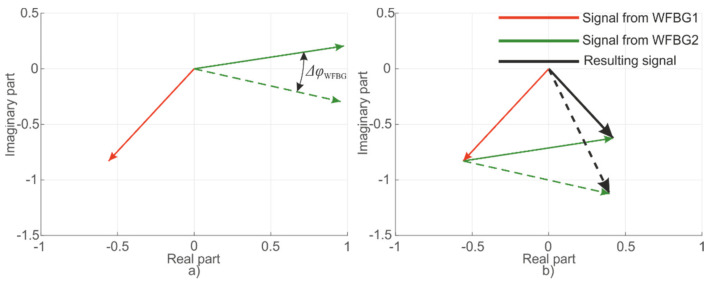
Signal conditioning scheme from two WFBGs: components (**a**) and summation (**b**).

**Figure 9:**
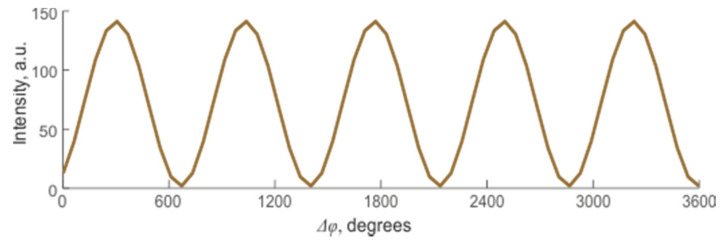
Change in the resulting intensity of the back-reflected signal depending on the distance between adjacent WFBGs.

For WFBGs, interference occurs between two waves reflected from adjacent structures. In this case, any periodic or monotonic fiber deformations between them will change the signal from its minimum to its maximum value. Figure 8 shows reflected waves from the first WFBG1 (*φ*_1_ = const) and the second WFBG2 (*φ*_2_ = var) as well as the resulting wave. These plots are presented on a complex plane, with the real and imaginary components of the light wave plotted along the abscissa and ordinate, respectively, and the arrows denoting the added backscattered components. Their modulus is equal to the wave amplitude, and the angle with the abscissa axis is the current phase. In this case, several backscattered waves can be added to the resulting vector according to the rules of vector addition. A linear change in length between two adjacent WFBGs results in a harmonic change in the sum signal, as shown in Figure 9, which represents the change over time in the resulting vector’s amplitude.

Mathematically, this can be expressed as the sum of two waves. The amplitude difference can be neglected because it will be no more than 1% between neighboring gratings.

(5) $$E^{2}=E_{1}^{2}+E_{2}^{2}+2E_{1}E_{2}cos(\varphi_{2}-\varphi_{1})$$

To describe the formation of a back-reflected signal from neighboring WFBGs, we can take *φ*_1_ as a constant because the deformations in the measured section’s fiber will not affect the phase difference of the signal in the section itself. In this case, the phase of the second grating due to the effect changes by the value Δ*φ_WFBG_*:

(6) $$E^{2}=E_{1}^{2}+E_{2}^{2}+2E_{1}E_{2}cos\left(\left(\varphi_{2}+\Delta \varphi_{WFBG}\right)-\varphi_{1}\right).$$

This expression shows that with a linear phase change, the system signal will change as a function of the cosine. In this case, the magnitude of the phase change itself will be determined by the overall change in the length of the fiber between the gratings:

(7) $$\Delta \varphi_{WFBG}=\frac{4\pi \Delta L}{\lambda }.$$

Finally, we can formulate common requirements for WFBGs for phi-OTDR interrogation in the Table 1.

## 3. Calculation

### 3.1. Calculation of the Signal and Noise Level in a Phase-Sensitive Reflectometer

As mentioned above, the main components generating noise in this system are laser wavelength instability, optical preamplifier noise, and photodetector module noise. A detailed analysis of the influences of instability in the laser source’s radiation is given in [18,56]. They can only be eliminated when using an ideal laser source or a source with negligible frequency fluctuations. The existing sources are not ideal, but their noise level is low enough not to consider this type of noise as the main one.

The noise of a photodetector module is determined by the quality and technology of its manufacturing. It is proportional to the specific equivalent noise power A and the root of the frequency band ∆*f* recorded by the radiation receiver, which, in turn, is determined by the spatial resolution of the system ∆*z*. Using the average value Δ*z* = 20 m, attained at a pulse duration *τ* = 200 ns, the minimum required frequency band recorded and orbited by the photodetector module will be ∆*f* = 1/*τ* = 5 MHz. Higher frequency ADCs are often used when increasing the number of points for processing and detection, from 50 to 100 MHz. This makes it possible to increase the sampling resolution but keeps the spatial resolution the same.

The scheme of phi-OTDR uses a preamplifier generating the third type of noise in question to boost the extremely low backscattered signal level. With a pulse duration *τ* = 200 ns, the backscattered fraction of the pulse is approximately 10^−6^, following the expression

(8) $$r=r_{1}+10\log\left(\tau \right)=-80+10\log\left(200\right)=-57\;\mathrm{dB},$$

where *r*_1_ = −80 dB—is the power fraction scattered by a 1 ns pulse.

This value is the most frequently encountered when creating monitoring systems for extended objects. Increasing it will give a higher level of received power but will worsen the spatial resolution. The power of the signal coming from the *L_S_* km sensor can be obtained from the expression

(9) $$P_{sig}=P_{in}+\eta +2\alpha L_{S}+r,$$

where *P_in_* = 23 dBm (the upper value is limited by nonlinear effects [17]) is the peak pulse power, *η* = −4 dB is the loss at connectors and elements, *α* = −0.18 dB/km is the fiber-attenuation coefficient, and *L_S_* is usually no more than 50 km. That is, *P_sig_* ranges from 146 nW (nearby sites–less than 1 km) to 5.8 nW (distant sites–about 40 km).

The magnitude of the generated signal-spontaneous noise can be determined by the expression [53]

(10) $${\sigma_{s-sp}}^{2}=2G^{2}P_{sig}NFh\nu \Delta f,$$

where *G* = 1000 is the preamplifier gain, *P_sig_*, nW is the amplified signal power, *NF* = 4 is the preamplifier noise factor, *h* = 6.626∙10^−34^ J∙s is the Planck constant, *ν* = 193.4 THz is the radiation frequency, and ∆*f* = 5 MHz is the receiver frequency bandwidth. The value of *σ_s−sp_* ranges from 0.9 (nearby sections) to 0.2 μW (distant sections). In this case, the signal power will range (after amplification by a factor of *G*) from 150 (nearby section) to 6 μW (distant section). Note that the preamplifier also generates spontaneous-spontaneous noise *σ_sp−sp_*

(11) $$\sigma_{sp-sp}{}^{2}={\left(NFh\nu G\right)}^{2}2\Delta f\left(\Delta \nu -\Delta f/2\right)\approx {\left(36\mathrm{nW}\right)}^{2},$$

where ∆*ν* is the optical filter’s bandwidth. When using better and more expensive samples with ∆*ν* = 1 GHz, this value is two orders of magnitude less than the beat noise of signal radiation with spontaneous radiation. This value will be higher when using the more common and less expensive filters with ∆*ν* = 12.5 GHz, but still will have an order of magnitude less signal-spontaneous noise. It is impossible to not use a preamplifier in the standard scheme. Without one, only signals from the first several kilometers of the sensor could be measured, with the help of existing radiation receivers. The photodetector’s noise level is determined by the frequency band and the equivalent noise power (a receiver characteristic), and its value is similar to that of the level of spontaneous-spontaneous noise *σ_pd_* = 10 nW.

### 3.2. Calculation of the Signal and Noise Levels in the WFBG System

The WFRB system does not use an optical preamplifier, and the main parameter for calculating the signal power is the gratings’ reflection coefficient. The expression can be written as follows

(12) $$P_{WFBGsig}=P_{in}{\left(1-R\right)}^{2(N-1)}exp(-2\alpha_{WFBG}L_{S})R,$$

where *P_in_*, W is the peak power, *α_WFBG_*, km^−1^ is the fiber attenuation with WFBGs, *N* is the number of gratings per 1 km, *L*, km is the sensor length, and *R* is the reflectance of one WFBG.

In this case, the peak pulse power is not limited by nonlinear effects—since the gratings’ reflection coefficient is orders of magnitude higher than the Rayleigh scattering value—but by the dynamic range of the receiving part’s intensity, in particular the ADC. Studies were carried out on the same components for both the phi-OTDR and the WFBG system. Therefore, the parameters of the peak pulse power were selected to fall into the same power range. The maximum (from the nearby site) was *P_WFBGsig_* = 150 μW. For this, given the assembled experimental scheme based on a sensor fiber with parameters *R* = 0.003, *N* = 50, it was necessary to inject 70 mW pulses into the sensor following Equation (12). In this case, the main source of the noise was the photodetector or the laser source’s instability, as in the experiment with a phase-sensitive reflectometer, *σ_pd_* = 10 nW. The effect of shifting the radiation wavelength from *λ* to *λ* + Δ*λ* can be estimated from Expression (6)

(13) $$\Delta \varphi_{WFBG}=\frac{4\pi L}{\lambda }-\frac{4\pi L}{\lambda +\Delta \lambda }=4\pi L\frac{\Delta \lambda }{\lambda \left(\lambda +\Delta \lambda \right)}\approx 4\pi L\frac{\Delta \lambda }{\lambda^{2}}=4\pi L\frac{\Delta \nu_{las}}{c},$$

where *L* is the distance between the gratings, *c* is the speed of light, and Δ*𝜈_las_* is the width of the laser line. We used a BasikX15 laser with a linewidth of less than 300 Hz and a grating spacing of 20 m, which provided an estimated phase shift of less than 0.085 mrad—that is, less than 0.003% of the maximum intensity fluctuation value of π rad. The noise level created by the laser source’s frequency instability will be less than *σ_las_* = 0.003%(*P_WFBGsig_*) = 4.5 nW, which, in this case, is comparable to the receiver’s noise level. However, note that many laser sources have a wider bandwidth, and this type of noise will become the main one in the system.

In this way, the increase in signal-to-noise ratio can be estimated based on the decreasing noise level while the emission carrier’s power is maintained. This value will be

(14) $$SNR_{enh}=\frac{\sigma_{s-sp}}{\sqrt{{\sigma_{pd}}^{2}+{\sigma_{las}}^{2}}}=\frac{900\mathrm{nW}}{11\mathrm{nW}}=82\;times\approx 19\;\mathrm{dB}.$$

However, the useful signal from sensors of this type is not the general power level but its deviation when the effect appears. In addition, because the signal itself and the response to it are non-stationary in time, it is easier to obtain information based on a statistical characteristic, specifically the RMS of the signal section’s intensity in the time interval corresponding to the effect on the fiber. The ratio of the RMS of the section with the signal to that of the section without the signal is taken to determine the signal-to-noise ratio.

## 4. Results

For the experiments, two setups were assembled: a phase-sensitive reflectometer utilizing backward Rayleigh scattering and a schematic of a WFBG reflectometer with a WFBG reflection coefficient *R* = 3 × 10^−3^ and *N* = 50 gratings per km (Figure 1 and Figure 5, respectively). We were interested in SNR enhancement from the nearby site (<1 km). The values for other distances and periodicities of the gratings’ arrangement can be determined using the calculations in [35,36].

The amplitude of the recorded signal was tested using cylindrical piezoelectrics, on which a sensor fiber was wound in one layer without overlaps. For a conventional phi-OTDR, the length of the wound cable was 22 m. This distance does not violate the equivalence to the sensitive area of the WFBG-based system, which is equal to the distance between the gratings (20 m) since the resolution of the reflectometric system is determined only by the pulse duration Δ*z* = *cτ/(*2*n)*, and at *τ* = 200 ns, it will also be 20 m. This value makes it possible to guarantee that at least one segment, which is entirely interrogated by the probe pulse, will fall into the phi-OTDR trace, almost without increasing the area of influence on the fiber. The pulse parameters are given in Table 2. For a WFBG-based OTDR, a section between adjacent WFBGs was wound on a piezoelectric cylinder. The pulse length was chosen to guarantee the interference of this pair of gratings without the neighboring ones being captured.

The nearby section of the fiber (within the first kilometer) was tested. The power of the optical amplifiers was adjusted to obtain a signal in the tested section at a peak level of 150 μW, as used in the previous calculations. The main goal was to analyze the magnitude of the improvement in the signal-to-noise ratio in this section when switching from a phase-sensitive reflectometer to a system based on WFBGs. The transition to the calculated sensitivities in the distant sections of the sensor can be found based on the expressions given in the calculation part of this work.

**Figure 10:**
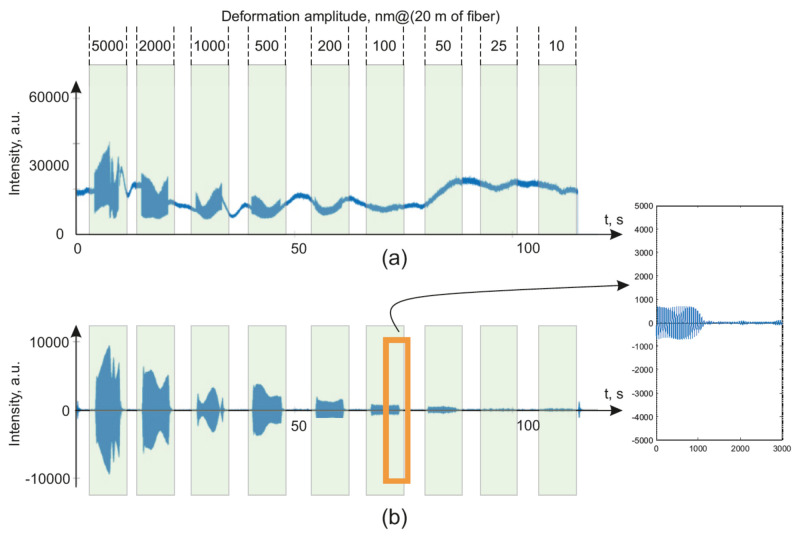
Initial data from the signal of a phase-sensitive reflectometer utilizing backward Rayleigh scattering when exposed to 20 Hz (**a**) and filtered for frequencies of 18–22 Hz (**b**) with zoomed data for 100 nm amplitude impact.

**Figure 11:**
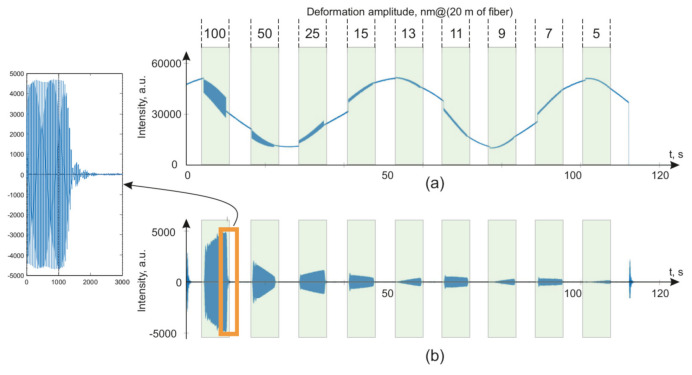
Initial data from the signal of a reflectometer based on WFBG with a pulse duration of 300 ns (provides spatial resolution 20 m when the distance between WFBGs is equal 20 m) when exposed to 20 Hz (**a**) and filtered for frequencies of 18–22 Hz (**b**) with zoomed data for 100 nm amplitude impact.

The signals received from the installations are plotted in Figure 10 for φ-OTDR and in Figure 11 for WFBG-OTDR. The impact was carried out with different amplitudes at a 20 Hz frequency. The maximum strain values are shown on the graph opposite the corresponding system response.

The presented graphs show the main differences in the general forms of the signals from the backward Rayleigh scattering reflectometers versus those based on WFBGs. The signal from the WFBGs changes smoothly, in the form of a sinusoidal curve, due to the monotonous temperature deviation of the sensor, as shown in the theory section. The signal from a backward Rayleigh scattering reflectometer has a complicated envelope due to the interference of backscattered waves from a huge number of scattering centers randomly located in the fiber. For the filtered signals, the signal-to-noise ratio was determined as the ratio of the root–mean–square deviation (RMS) of the area’s signal to the impact of the RMSD for the signal in the unimpacted area. A comparison of RMSD values is more useful for detection algorithms applications [57,58,59] than a comparison of spectral peak’s level [46]. The data were filtered with a second-order Butterworth bandpass filter in the range from 18 to 22 Hz. In this case, all of the sensor readings were passed through the filter in the range of ±50 m from the coordinates at which the action was generated, after which the sample was selected at which the deflection caused by fiber deformation had the largest maximum amplitude. The readout from the impact coordinates deviated by no more than ±5 m.

**Figure 12:**
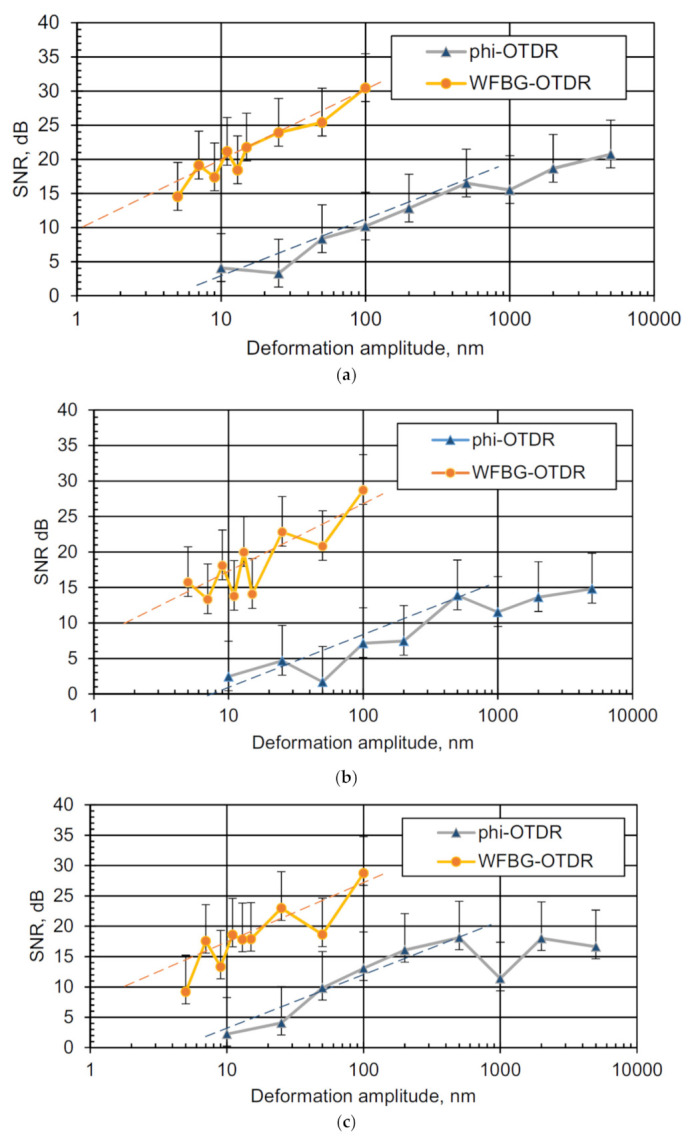
Graphs of the SNR for OTDRs using backscattering and based on WFBGs when recording the impacts on the fiber with different deformation amplitudes at (**a**) 20 Hz, (**b**) 100 Hz, and (**c**) 400 Hz frequencies. Dashed lines—trends of SNR dependence from amplitude.

The frequency of 20 Hz was chosen because it is one of the most important frequencies for recording vibration signals in the ground [60,61]. The following values of the signal-to-noise ratios were obtained for this frequency, as presented in the graph in Figure 12a.

The difference between the graphs is slightly less than two orders of magnitude, which is consistent with the previously calculated value *SNR_enh_*. The sensitivity study for two other frequencies—100 Hz (the upper limit of the propagated waves in ordinary soil media) and 400 Hz (vibrational waves propagating in ice and rocks)—are shown in Figure 12b,c, respectively.

As in the case of exposure at a frequency of 20 Hz, the WFBG-based system also shows a higher signal-to-noise ratio at frequencies of 100 and 400 Hz, and therefore greater sensitivity, by about 1.5–2 orders of magnitude, as compared to the classical phi-OTDR scheme. Trend lines show that the 10 dB SNR threshold for the WFBG-OTDR system is located in the impact range from 1 to 2 nm, which is also 1.5–2 orders better than the phi-OTDR scheme. For phi-OTDR, without phase reconstruction, SNR growth stops near 1000 nm impact amplitude due to signal wrapping.

## 5. Discussion

In this work, the signal formation was analyzed in a phase-sensitive reflectometer and a system based on WFBGs. On this basis, the main noise components in each scheme were determined, and the theoretical value of the sensitivity increase in the WFBG system was calculated. In our calculations, we used the assumption that the recorded return signal should be into the same range in intensity for phi-OTDR and WFBG-OTDR systems, and only the system’s noise is present without background external influences on fiber. This was proposed to calculate the near zone of the cable, where the attenuation in the sensor fiber can be neglected. These assumptions allow an estimate of the signal-to-noise improvement for fiber with WFBG. The reduction in the desired signal from attenuation can be performed for each specific fiber based on its parameters. The experimental results show that the calculated values agree within the error limits, mainly due to the random nature of scattering in the sensor of a phase-sensitive reflectometer. For a test fiber sample with basic parameters R = 0.003, N = 50 gratings per km, the total noise decreased by about 19 dB, which allows a WFBG-OTDR to register weaker or distant impacts on the sensor.

## Figures and Tables

**Table 1:** Common parameter of WFBG for phi-OTDR.

Parameter	WFBG-OTDR
Reflectivity, %	From 0.001 to 1, depending on needed sensor length [35]
Reflectivity instability (relative), %	<15
Central wavelength	Similar to the laser source of the system
Central wavelength instability	Less than 10% of WFBG spectral width
Spectral width, nm	Better, not less than 1 to avoid temperature and strain influence on the reflectivity spectrum. Fiber optic cable which is usually used for sensor installation provides some protection from external damaging influences, but some wavelength shifts due to seasons of the year or slow ground movements are still possible. The wide spectrum of WFBG reduces their effect on reflectivity changes and makes easier the process of laser wavelength and reflectivity spectrum matching.

**Table 2:** Parameters of experimental setups.

Parameter	φ-OTDR	wFBG-OTDR
Resolution, m	20 (determined by the duration of the probe pulse)	20 (determined by the distance between the gratings)
Fiber length at PZT, m	22	20
Fiber strain range at PZT, nm	From 10 to 5000	From 5 to 100
Range of supplied frequencies, Hz	20, 100, 400	20, 100, 400
